# Brief smoking cessation advice from practice nurses during routine cervical smear tests appointments: a cluster randomised controlled trial assessing feasibility, acceptability and potential effectiveness

**DOI:** 10.1038/sj.bjc.6603684

**Published:** 2007-04-03

**Authors:** S Hall, E Reid, O C Ukoumunne, J Weinman, T M Marteau

**Affiliations:** 1 Department of Palliative Care, Policy and Rehabilitation, School of Medicine at Guy's, King's College and St Thomas' Hospitals, King's College London, London; 2 King's College London, Institute of Psychiatry, Department of Psychology, Health Psychology Section, 5th Floor, Thomas Guy House, Guy's Campus, London SE1 9RT; 3 Clinical Epidemiology and Biostatistics Unit, Murdoch Childrens Research Institute and Department of Paediatrics, University of Melbourne, Australia

**Keywords:** smoking cessation, primary health care, nurse's role, primary prevention, uterine cervical neoplasms

## Abstract

The aim of this study is to assess the potential effectiveness, acceptability and feasibility of a brief smoking cessation intervention delivered as part of cervical screening. A cluster randomised controlled trial was conducted with clinic week as the unit of randomisation, comparing a group (*n*=121) receiving brief smoking cessation advice supplemented with written information given by practice nurses during cervical smear test appointments, with a group (*n*=121) not receiving this advice. Outcomes were intention to stop smoking (potential effectiveness); intention to attend for future cervical screening (acceptability); duration of intervention (feasibility). 172/242 (71%) and 153/242 (63%) participants completed 2-week and 10-week follow-ups, respectively. Compared to women in the control group, those in the intervention group had higher intentions to stop smoking at 2-weeks (adjusted mean difference 0.51, 95% CI: −0.02 to 1.03, *P*=0.06) and 10-weeks (adjusted mean difference 0.80, 95% CI 0.10 to 1.50, *P*=0.03). The two groups had similarly high intentions to attend for future screening. Consultations in the intervention arm took a mean of 4.98 min (95% CI: 3.69 to 6.27; *P*<0.001) longer than the control arm. In conclusion, brief smoking cessation advice given by practice nurses as part of cervical screening seems acceptable, feasible and potentially effective. Evidence is lacking on the effectiveness and cost effectiveness of this intervention in achieving biochemically validated smoking cessation.

A report from the International Agency for Research on Cancer from an Expert Group highlights the need for systematic study of the feasibility of combining routine screening with health education and its consequences as part of their proposed strategy for the development of research in the behavioural, social and related sciences relevant to cancer (IARC, 2004). Smoking is a risk factor for many diseases including some in which screening is available. These include cervical cancer, colorectal cancer, heart disease and abdominal aortic aneurysms. Screening provides an opportunity to inform smokers of the health risks of smoking and to motivate smoking cessation. However, evidence is needed to show that such interventions are effective and that they do no harm, such as deterring people from re-attending for screening. The aim of the current study is to generate preliminary evidence regarding the impact of providing brief smoking cessation advice as part of screening for cervical cancer.

Smoking doubles the chance of developing cervical cancer (Szarewski and Cuzick, 1998) and is an independent predictor of treatment failure in women with cervical intraepithelial neoplasia (Acladious *et al*, 2002). Stopping smoking can result in low-grade lesions remitting within 6 months (Szarewski *et al*, 1996); many women are unaware of this (Marteau *et al*, 2002). Written information about the link between smoking and cervical cancer increases motivation to stop smoking (Hall *et al*, 2003, 2004; Bishop *et al*, 2005). The effectiveness of this information could be increased if combined with verbal smoking cessation advice (Lancaster and Stead, 2005). Although it has been suggested that smokers are less likely than non-smokers to attend for cervical screening (Orbell *et al*, 1995), evidence concerning this association is equivocal with other studies finding no association (Clark *et al*, 2000). Since 81% of women aged between 25 and 64 in England participate in the cervical screening programme (Patnick, 2003), this time provides an ideal opportunity to deliver effective smoking cessation interventions to reduce the high rates of smoking in women, reducing risks of cervical cancer as well as other smoking-related diseases.

Brief smoking cessation advice from physicians is effective (Lancaster and Stead, 2004). A Cochrane review of nurses' smoking cessation interventions shows that nurses are effective in motivating smokers to quit. These interventions, however, were generally more intensive than those given by physicians and the evidence is not strong for an effect when it is provided as part of a health check in general practice (Rice and Stead, 2004). We are unaware of any suitably powered studies in primary care evaluating the impact of brief, opportunistic smoking cessation advice delivered by nurses. It is also important to ensure that giving unsolicited smoking cessation advice does not annoy women (Butler *et al*, 1998) or deter them from attending for future cervical screening. Such direct evidence is needed to inform the recent policy in the UK which states that nurses in primary care should deliver brief smoking cessation advice to everyone who smokes (National Institute for Clinical Excellence, 2006). The current pilot study assesses the potential effectiveness, acceptability and feasibility of a brief smoking cessation intervention delivered by nurses in the context of a cervical screening consultation, to inform the decision about the need for a clinical trial.

## MATERIALS AND METHODS

### Participants

**Figure 1:**
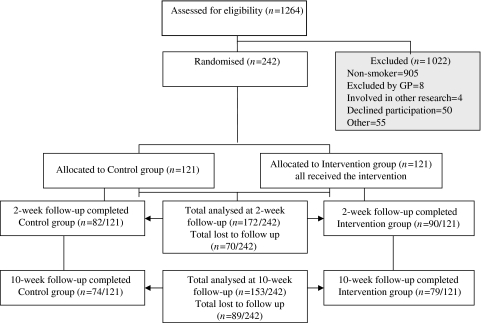
Movement of women through the study.

The study population consisted of smokers attending for cervical screening between January and December 2004. Women were invited for screening as part of the national cervical cancer screening programme. One practice nurse, currently conducting cervical smear tests from each of eight general practices located in the south east of England that are part of the Medical Research Council General Practice Research Framework, participated in the study. Women were eligible to participate if they smoked at least one cigarette a day and understood spoken English. We excluded women from the study if they were participating in any other intervention study (see Figure 1). In addition, GPs checked weekly lists of eligible women and excluded those whom they judged should not be approached.

### Ethical approval

The Metropolitan Multi-Centre Research Ethics Committee (03/11/067) and all relevant local research ethics committees approved the study.

### Randomisation

Clinic weeks (clusters) were the units of randomisation, with all women attending for a smear test at the study practices in a given week allocated to the same trial arm (Donner and Klar, 2000). We considered two other units of randomisation for this study: (a) cluster randomisation with GP practices as the unit of randomisation and (b) randomisation by patient. With a larger study (20 or more practices), cluster randomisation by GP practice would have been preferred. However, with just eight practices, it would not have been possible either to estimate precisely the intra-cluster (intra-practice) correlation coefficient at analysis or to use adjusted individual-level methods (Ukoumunne *et al*, 1999). Randomisation by patient would have avoided this issue, but, because nurses would have frequently had to switch from intervention to control procedures, this would have had the highest likelihood of contamination between the trial arms. We therefore chose randomisation by clinic week because, with relatively few women attending each week, the variance inflation factor (Ukoumunne *et al*, 1999) was likely to be small and the likelihood of contamination was at least reduced, since nurses were instructed at the end of the preceding week to take only the study packs for either the intervention or the control arms for the following week. Clinic weeks were randomly allocated by the study statistician (OU) to the trial arms using computer-generated random numbers. Block randomisation (Roberts and Torgerson, 1998) was used with three different random block sizes of two, four and six to prevent long sequences of clinic weeks being randomised to the same trial arm. At the end of each week during the recruitment period, the study coordinator (SH) informed nurses whether the following week would be an intervention or a control week. As a consequence of these arrangements, there was no allocation concealment.

### Procedure

The receptionist gave all women attending for routine cervical smear tests information to read about the study while they were waiting to see the nurse. This clearly stated that the information was only relevant to smokers. Before conducting the smear test, nurses asked women if they smoked. If women reported that they smoked, nurses checked their understanding of the information sheet, provided clarifications where necessary and then invited them into the study.

During intervention weeks, nurses delivered brief smoking cessation advice as part of the smear test visit to all smokers who consented to take part in the study. In control weeks they did not give smoking cessation advice.

### The intervention

Nurses delivered the intervention to each participant just once. The intervention was designed to take about 3 min to deliver. We based the advice on the ‘5 As’ designed for health professionals assisting patients in stopping smoking (*A*sk, *A*dvise, *A*ssess, *A*ssist and *A*rrange) for which there is evidence of effectiveness (West, McNeill and Raw, 2000). As recommended in The Expert Patient Programme (Department of Health, 2001), we trained nurses to give advice in a way that builds rapport and is patient-centred. In addition, nurses gave all women in the intervention group an information pack containing: (a) the leaflet we developed for the study: *Smoking and Women's Health*; (b) the self-help booklet produced by the Department of Health: *Giving up for life*; (c) the booklet produced by QUIT: *Quit smoking without putting on weight*; and (d) a card listing local and national smoking cessation services.

All nurses received one and a half days of training in giving smoking cessation advice. Medical Research Council General Practice Research Framework regional training nurses visited each nurse to undertake a structured quality control assessment to ensure nurse compliance with the protocol.

### Measures

We assessed outcomes by postal questionnaire 2 weeks and approximately 10 weeks after the consultation. We sent most women questionnaires 10 weeks after recruitment. However, if test results had not been sent by 10 weeks, we delayed sending the second questionnaire until a week had passed since test results had been sent. Nine women chose to complete these measures by telephone. We sent one reminder to those who had not responded within 2 weeks.

Since this study is aimed at providing a ‘proof of principle’, our main outcome is intention to stop smoking in the next month (potential effectiveness). We used two, seven-point scales at the 2- and 10-week follow-ups. As these produced a reliable measure (Cronbach's *α*=0.81 at 2 weeks and 0.91 at 10 weeks), we used the mean of the two items in the analyses. We also collected self-reported smoking status. We assessed the feasibility of the intervention by estimating the time taken for the intervention. We assessed the acceptability of the intervention to women by measuring intention to return for next cervical smear test appointment using a single, seven-point scale. As this measure was highly skewed we dichotomised it (definitely intending: score 7, *vs* other: scores 1–6). Baseline readiness to stop smoking (contemplators: planning to stop within the next 6 months; pre-contemplators: not planning to do so) and demographic measures was collected by nurses before the intervention. Results of current smear tests were obtained from practice nurses.

### Sample size

We planned to detect an effect size of 0.42 standard deviation units in mean intention to stop smoking in the next month between the intervention and control groups, with 80% power at the (two-tailed) 5% level of significance. This is the effect size found in our leaflet evaluation (Hall *et al*, 2004), and corresponds to a medium effect (Cohen, 1988). In a trial randomising individuals, 89 participants would be required in each trial group. After inflating this sample size by 10% to allow for a possible variance inflation factor resulting from the randomisation by clinic weeks rather than individual participants (Ukoumunne *et al*, 1999), and a further 30% to allow for loss to follow-up, a total of 288 participants are required. We planned to recruit 36 smokers from each of the eight participating general practices.

### Statistical analysis

Analyses were conducted using the intention to treat principle as far as possible, given missing data. Analyses of drop-out status were implemented. Intention to stop smoking and length of smear test consultation were compared between trial arms using random effects linear regression models (Goldstein, 1995) fitted using restricted maximum likelihood estimation to allow for clustering of participant responses within randomised weeks. As these measures were skewed, the non-parametric bootstrap (Davison and Hinkley, 1997) was used to validate the confidence intervals, resampling weeks (clusters) rather than individuals. As the bootstrap confidence intervals were similar to the model-based intervals, we report the latter. Comparison of the 2- and 10-week outcomes were adjusted for age, ethnic group, education level, baseline motivation to stop smoking and whether the participant had previously had an abnormal cervical smear test result. Comparison of the 10-week outcome was additionally adjusted for whether the index smear result was normal. As few women reported stopping smoking, and most reported definitely intending to return for future smear tests, it was not possible to adjust these analyses for confounders or clustering. We conducted Fisher's exact tests for these dichotomous outcomes. All analyses were carried out using Stata 9.2 (StataCorp, 2005).

## RESULTS

The final sample comprised 121 women in each trial arm (Figure 1). The GPs excluded eight women. The proportion of women who declined to participate did not differ between the intervention (31 out of 152, 20%) and control weeks (19 out of 140, 14%) (*χ*^2^=2.39, d.f.=1, *P*=0.12). Baseline and demographic characteristics of the intervention and control groups are shown in Table 1. About 26 (31 out of 121) and 32% (39 out of 121) participants in the intervention and control arms, respectively, were lost to follow-up at 2 weeks. About 35 (42 out of 121) and 39% (47 out of 121) participants in the intervention and control arms, respectively, were lost to follow-up at 10 weeks. Non-responders at the 2- and 10-week follow-ups were younger, and non-responders at the 10-week follow-up were more likely to have received a normal result on their current smear test. Smokers educated to GCSE ‘O’ level standard or less were more likely to be lost to follow-up if they were randomised to the control group than their counterparts in the intervention group. None of these variables, however, was associated with intention to stop smoking.

### Outcomes

The outcomes are summarised in Table 2. Compared to women in the control group, those in the intervention group had higher intentions to stop smoking, both at the 2- (*P*=0.06) and 10-week (*P*=0.03) follow-ups. More women in the intervention arm than the control arm reported smoking cessation at 10 weeks (12 *vs* 5, *P*=0.13), but the difference was not significant at the 5% level. Intentions to attend for next cervical smear test were high at 2 weeks and 10 weeks in both trial arms. The mean time taken to complete the smear test consultation was longer in the intervention arm than in the control arm (mean difference 4.98 min; 95% CI: 3.69–6.27; *P*<0.001).

## DISCUSSION

This study suggests that a brief smoking cessation intervention given by trained practice nurses as part of routine cervical screening is acceptable, feasible and has the potential to motivate women to stop smoking without deterring them from attending for future cervical screening.

A priority in developing this intervention was to ensure that it was feasible to incorporate it into routine cervical screening appointments, and that it did not have the unintended effect of deterring women from attending for future cervical screening. The intervention took longer to deliver than the planned 3 min. This may reflect women's interest in the link between smoking and cervical cancer and their willingness to discuss their smoking with these nurses trained to give advice in a patient-centred manner. Although it has been suggested that unsolicited smoking cessation advice can annoy patients (Butler *et al*, 1998), a qualitative study of 16 women in the intervention group of the current study found that they raised no objections to being giving smoking cessation advice from the trained nurses. The results of the current study also show that providing such advice does not diminish intentions to return for future cervical smear tests.

In addition to doing no harm, the intervention increased women's intentions to stop smoking in the next month evident both at 2- and 10-week follow-ups. This raises two questions: (i) how effective is the intervention likely to be in achieving actual quitting, and (ii) is there sufficient uncertainty in this answer to justify an RCT? Objectively measured behaviour, in this case, biochemically validated smoking cessation, is a harder outcome than intentions to stop smoking in the next month and, where appropriate (e.g. in the conduct of a clinical trial), they are the end point of choice. The current study did not use behaviour as the end point as it was designed to assess the acceptability, feasibility and potential effectiveness of the intervention before a clinical trial. Nonetheless, intentions are a reliable predictor of health-related behaviour, including smoking (Armitage and Connor, 2001). Intentions not to engage in a behaviour are highly predictive of not doing so, whereas intentions to engage in a behaviour are less reliable (Sheeran and Orbell, 1998). Thus, if a smoking cessation intervention has no impact on intentions to stop smoking, it is highly unlikely that such an intervention will achieve smoking cessation. If, however, an intervention does increase intentions to stop smoking, then it is possible that it could result in smoking cessation. It is therefore informative to use intentions to stop smoking as an end point for a study designed to assess the potential effectiveness of an intervention. Failure to find an impact upon intentions would avoid the costs of conducting a trial powered on smoking cessation as the end point. In keeping with the MRC framework for evaluating complex interventions, the current study can be seen as collecting theoretical and empirical evidence for the likely effectiveness of the intervention (Medical Research Council, 2000).

We cannot judge with much confidence the size of effect the current intervention is likely to have in achieving smoking cessation. We believe that the results of this pilot study warrant a randomised controlled trial to evaluate the intervention for its effectiveness and cost-effectiveness at achieving biochemically validated smoking cessation (estimated to involve 7200 smokers), given the importance of developing cost-effective smoking cessation interventions (World Health Organization, 2003), the uncertainty attached to using intention as a proxy for actual behaviour change, and the opportunity cost of delivering an ineffective intervention.

The main strength of this study is that, to the best of our knowledge, it is the first study in UK primary care to determine the impact of opportunistic brief smoking cessation advice from nurses when effective smoking cessation services are readily available. It is also the first to consider the possible harms of delivering such interventions as part of routine screening.

The main weaknesses concern: the main outcome measure, the response rates to the questionnaires and nurses not being blinded to group allocation. As described above, intentions to stop smoking are a proxy measure of smoking cessation. However, the aim of the study was not to detect a change in behaviour but rather to demonstrate the feasibility of a clinical trial as well as provide ‘proof of principle’ (Medical Research Council, 2000) of an innovative smoking cessation intervention. We are now arguing that the results of this pilot study support the case for a trial. The proportion of participants lost to 10-week follow-up was greater than expected. This introduces potential bias, the nature and scale of which is unknown. It underscores the need for future studies in this area to assess outcome measures using methods with higher response rates. These include participant payment and telephone administered questionnaires (McColl *et al*, 2001). Another weakness and potential source of bias in the current study is the lack of allocation concealment, that is the nurses knew at the time of recruitment the treatment arm to which women were to be randomised. This could have led to selection bias with different numbers of women with different characteristics being recruited during the intervention compared with the control weeks (Medical Research Council, 2002, pp 4–5). The fact that equal numbers of women were recruited to each arm suggests that this may not have been a serious problem in this trial. Women in the intervention arm were more likely to have had a previous abnormal cervical smear test result than those in the control arm (43 *vs* 32%), but otherwise there were no marked differences in demographic or baseline characteristics between the women in the two trial arms.

In conclusion, the results of this study suggest that brief smoking cessation advice given by practice nurses during routine cervical smear test appointments is acceptable, feasible and potentially effective. Evidence is lacking on the effectiveness and cost-effectiveness of this intervention in achieving biochemically validated smoking cessation. Such evidence is needed to inform current practice and health policies in the UK and elsewhere aimed at reducing deaths from smoking.

## Figures and Tables

**Table 1:** Baseline and demographic characteristics of the intervention and control groups

**Characteristic**	**Statistic**	**Intervention (*n*=121)**	**Control (*n*=121)**
Age	Mean (s.d.)	38.7 (11.8)	39.3 (12.1)
*Baseline stage of change*			
Pre-contemplation	*n* (%)	53 (44)	61 (50)
Contemplation	*n* (%)	67 (55)	60 (50)
Missing	*n* (%)	1 (1)	0 (0)

*Ethnic group*			
White	*n* (%)	113 (93)	111 (92)
Other	*n* (%)	8 (7)	10 (8)

*Education*			
GCSE ‘O’ level or less	*n* (%)	68 (56)	64 (53)
‘A’ level or higher	*n* (%)	53 (44)	57 (47)

*Previous cervical abnormality*			
Yes	*n* (%)	52 (43)	39 (32)
No	*n* (%)	66 (55)	82 (68)
Missing	*n* (%)	3 (2)	0 (0)

*Current smear test result*			
Normal	*n* (%)	97 (80)	96 (79)
Abnormal/borderline	*n* (%)	24 (20)	23 (19)
Inadequate/unclear	*n* (%)	0 (0)	2 (2)

**Table 2:** 2- and 10-week outcomes by randomisation group

**Outcome**	**Intervention**	**Control**	**Difference**	**Adjusted mean difference 95% CI**	***P*-value**
2-week follow-up	*n*=90	*n*=82			
					
Stopped smoking, *n* (%)	3 (3)	2 (2)			1.00
Intention to stop smoking in the next month, mean (s.d.)	2.86 (1.80)	2.29 (1.71)	0.51	−0.02 to 1.03	0.06
Definitely intend to go for next cervical smear test, *n* (%)	86 (97)	74 (90)			0.12
					
10-week follow-up	*n*=79	*n*=74			
					
Stopped smoking, *n* (%)	12 (15)	5 (7)			0.13
Intention to stop smoking in the next month, mean (s.d.)	3.13 (2.04)	2.24 (1.58)	0.80	0.10–1.50	0.03
Definitely intend to go for next cervical smear test, *n* (%)	74 (94)	70 (95)			1.00

Sample size for adjusted analysis of intention to stop smoking was 83 in the intervention arm and 77 in the control arm at 2 weeks, and 65 in the intervention arm and 67 in the control arm at 10 weeks.
